# A meta-analysis of case–control studies on the combined effect of hepatitis B and C virus infections in causing hepatocellular carcinoma in China

**DOI:** 10.1038/sj.bjc.6602333

**Published:** 2005-02-01

**Authors:** J Shi, L Zhu, S Liu, W-f Xie

**Affiliations:** 1 Department of Gastroenterology, Changzheng Hospital, Second Military Medical University, Shanghai 200003, PR China

**Keywords:** liver neoplasms, hepatitis B virus, hepatitis C virus, case–control study

## Abstract

We investigated whether concurrent infection by hepatitis B virus (HBV) and hepatitis C virus (HCV) in China, a hyperepidemic area for these infections, was associated with a higher risk of causing hepatocellular carcinoma (HCC) than each infection alone in a meta-analysis in China, 32 case–control studies involving 3201 cases and 4005 controls, identified from a computer-based literature search from 1966 to 2004. The pooled odds ratio and 95% confidence interval (CI) for HBsAg positivity was 14.1 (95% CI: 10.6–18.8); for anti-HCV/HCV RNA positivity was 4.6 (95% CI: 3.6–5.9); for HBsAg positivity and anti-HCV/HCV RNA negativity were 15.6 (95% CI: 11.5–21.3); for HBsAg negativity and anti-HCV/HCV RNA positivity were 8.1 (95% CI: 5.0–13.0); and positivity for both HBsAg and anti-HCV/HCV RNA was 35.7 (95% CI: 26.2–48.5). We conclude that HBV and HCV infections are important independent risk factors for HCC in China, and that dual infection by HBV and HCV is associated with a higher risk of causing HCC than each infection alone, suggesting a synergism between HBV and HCV.

Hepatocellular carcinoma (HCC) is the fifth most common malignant neoplasm in the world, and the third most common cause of cancer-related death (Parkin *et al*, 2001). More than 500 000 new cases are currently diagnosed yearly, with an age-adjusted worldwide incidence of 5.5–14.9 per 100 000 population (Llovet *et al*, 2003). About 110 000 persons die each year from HCC in China, which accounts for 45% of the deaths from HCC worldwide. China is also a hyperepidemic area for hepatitis B virus (HBV) infection (with an estimated carrier rate exceeding 10% in the general population) and hepatitis C virus (HCV) infection (with a prevalence of anti-HCV 3.1% of in the general population) (Xuezhong *et al*, 1999). HBV and HCV infections have each been shown to markedly increase the risk of developing HCC. In China, HBV has, for several decades, been the major cause of liver disease (Tang, 2001). Soon after the identification of HCV and the development of testing HCV infection, it has become increasingly evident that chronic HCV infection also plays an important role in chronic hepatic disease including HCC (Yano *et al*, 1993; Tsai *et al*, 1994). There have been several meta-analyses of the relation of HBV and HCV infections to HCC in China, but the relation of dual infection to HCC has not received similar attention. In fact, this is not rare in HCC patients and a higher morbidity in HCC patients with dual infection has been reported than in those with one infection (Benvegnu *et al*, 1994). The main difficulty in evaluating the relationship of dual infection to HCC is the rarity of concurrent infection in subjects without clinically evident liver disease. Only large case–control studies that include people unaffected by chronic liver diseases as controls can allow the interaction to be properly assessed. The aim of this meta-analysis is to assess the interaction between HBV and HCV infections among 32 case–control studies of HCC.

## MATERIALS AND METHODS

### Data sources

We searched the published Chinese literature from 1979 to February 2004 and Medline database from 1966 to February 2004 to identify case–control studies of the combined effect of HBV and HCV in causing HCC using the search textwords ‘hepatocellular carcinima’ and ‘HCV’ or ‘hepatitis non-A, non-B virus’. We also did a full manual search from bibliographies of selected papers. Studies were identified by two researchers independently and the two lists compared and discrepancies resolved. We also contacted the authors of studies containing relevant information who did not report their results suited to this analysis. Unpublished data were also accepted if an abstract was available and further information was obtained from the author. We contacted most of the journals on gastroenterology, hepatology and epidemiology in China for studies in press.

### Study criteria

(i) Each case–control study was required to have published details of the number of participants and age-adjusted or age-matched odds ratio (OR) and 95% confidence interval (CI). (ii) We included only the studies that used the following serological markers of chronic viral infections: HBsAg for HBV infection, and anti-HCV (tested with enzyme-linked immunosorbent assay, ELISA) or HCV RNA (detected by reverse transfusion polymerase chain reaction (RT–PCR) for HCV infection. When data were available on both anti-HCV and HCV RNA tests, we used only the former for classifying subjects as positive or negative for HCV infection. (iii) Only studies were included that selected HCC patients as cases and subjects without chronic liver diseases as controls; blood donors and patients on usual blood transfusion, regular haemodialysis or peritoneal dialysis were not considered eligible as controls. (iv) The studies were ineligible if HBsAg positive subjects were excluded from controls. (v) Studies with less than 20 cases were excluded, since they could not detect any case positive for both HBsAg and anti-HCV/HCV RNA. (vi) When more than one publication from the same study was available, only the final one was used.

### Data extraction

Data were independently extracted from each study using predefined forms, and disagreement was resolved by consensus. The following information was extracted: the area where studies were performed; number of cases and controls; mean age of case- and control-groups; type of controls; number of cases and controls for each category of HBV and HCV infections; the OR and 95% CI for each category of HBV and HCV infections. The most fully adjusted OR and 95% CI were extracted from each paper.

### Statistical methods

The pooled OR and 95% CI were calculated from raw study data using the Mantel–Haenszel method (fixed effect model) as modified by Robbins *et al* (1986) or the DerSimonian–Laird method (random-effect model) (DerSimonian and Laird, 1986). When there was significant heterogeneity, the random-effect model was used; otherwise the fixed-effect model was used. Evidence of heterogeneity in the estimate of effect was checked using the Breslow-Day test. A linear regression of the reported ORs, or the ORs calculated by us, was also performed by a fixed effect weighted-mean/linear-regression analysis, using the OR variances as weights. Synergism between HBV and HCV infections was assessed by the method of Rothman (1986). To determine the source of heterogeneity, we analysed subgroups of case–control studies according to the following characteristics: type of controls (hospital *vs* community controls); geographical area (higher incidence area for HCC *vs* lower, the cutoff incidence being 30 per 100 000 population). (Studies in Jiangsu, Zhejiang, Guangdong, Guangxi, Fujian, Hainan were classified as higher incidence, and studies in other provinces as lower).

## RESULTS

A total of 32 case–control studies satisfied the inclusion criteria (Xu *et al*, 1990; Gong *et al*, 1993; Zhang *et al*, 1993, 1998; Ye *et al*, 1994; Zhou *et al*, 1994; Fan *et al*, 1995a, 1995b; Pan *et al*, 1995; Wang *et al*, 1996a, 1996b, 1999; Guo and Yan, 1997; Lu, 1997; Lu and Hou, 1997; Ma *et al*, 1997; Shen *et al*, 1997; Yu *et al*, 1997; Zuo *et al*, 1997; Dai *et al*, 1998; Gao *et al*, 1998; Cui *et al*, 1999; Li *et al*, 1999, 2002; Yang *et al*, 1999; Zhao *et al*, 2000; Shi *et al*, 2001; Su *et al*, 2002; Wang, 2003; Wu *et al*, 2003; Zeng *et al*, 2003; Ding *et al*, 2004). Their basic characteristics are shown in Table 1 . A total of 31 articles were published in Chinese and one in English. They included a total of 3201 cases and 4005 controls. Most studies took subjects, as controls, who were of the same sex, lived in the same area or saw a doctor at the same hospital as the cases. Mean age was not reported in 14 studies, but in most of these, the disparity in age between cases and controls was less than 3 or 5 years. Most studies showed no difference in mean age or in sex between cases and controls.

The summary OR and 95% CI for positivity for both HBsAg and anti-HCV/HCV RNA in the studies overall are presented in Table 2 by geographical area and type of controls. The test for heterogeneity showed significant differences (*P*<0.001) between studies whether considered overall or in subgroups. In the higher incidence areas, the summary ORs for HBsAg positivity and for anti-HCV/HCV RNA positivity were slightly higher than those found in lower incidence areas. The OR for HBsAg positivity using community controls was significantly higher than that with hospital controls. The OR for anti-HCV/HCV RNA positivity using community controls was rather similar to that using hospital controls.

The OR and 95% CI for each HBsAg and anti-HCV/HCV RNA combination in case and control groups are set out in Table 3 and 4. Large differences were observed, most studies showing a strong and statistically significant association between each marker and HCC. A very low proportion of cases unexposed to either marker was found in one study, which gave exceptionally high OR estimates for both HBsAg and anti-HCV/HCV RNA (Zhao *et al*, 2000). The proportion of HCC patients with anti-HCV/HCV RNA positivity was 19.13% among HBsAg positive patients and 28.01% among HBsAg negative patients in the studies overall (*P*<0.001). The corresponding proportions for reports using community controls were 15.46 and 21.70% (*P*<0.01), respectively, and using hospital controls, 22.84 and 32.01% (*P*<0.01).

The summary OR for each HBsAg and anti-HCV/HCV RNA combination are reported in Table 5 . In the studies overall, the OR for HBsAg positivity in anti-HCV/HCV RNA negative subjects was 15.6 (95% CI: 11.5–21.3), the OR for anti-HCV/HCV RNA positivity in HBsAg negative subjects was 8.1 (95% CI: 5.0–13.0) and the OR for positivity for both markers was 35.7 (95% CI: 26.2–48.5). The corresponding ORs for studies using community controls were 19.9 (95% CI: 12.4–32.0), 11.1 (95% CI: 5.7–21.7) and 39.5 (95% CI: 22.9–68.1), respectively. The corresponding ORs for reports using hospital controls were 11.9 (95% CI: 7.9–17.8), 5.9 (95% CI: 3.1–11.4) and 44.9 (95% CI: 27.2–74.1). The corresponding ORs for studies in the higher incidence area were 14.6 (95% CI: 9.9–21.4), 8.1 (95% CI: 4.5–14.7) and 34.0 (95% CI: 23.4–49.5), and in the lower incidence area 17.3 (95% CI: 10.2–29.4), 7.8 (95% CI: 3.4–17.8) and 39.3 (95% CI: 22.9–67.6). Significant heterogeneity was found in the studies overall and in the subgroups (*P*<0.001).

## DISCUSSION

This study represents a quantitative assessment of published data on the role of chronic HBV and HCV infections in causing HCC in China. The studies included differed widely in their OR estimates for each infective marker. Even after grouping the studies by geographical area and type of controls, we found significant differences within subgroups. However, in almost all studies, we found a strong association between HCC and positivity for both HBsAg and anti-HCV/HCV RNA, with summary OR 14.1 for HBsAg positivity and 4.6 for anti-HCV/HCV RNA positivity. In 1994, Zhao *et al* (1994) reported a summary OR for HBsAg positivity in HCC in China of 13.43, rather similar to our finding, indicating that the strength of the association in China has not altered much in the last decade. In 1998, Donato *et al* reported a summary OR for HBsAg positivity in HCC worldwide of 13.7, little different from that in western countries.

In 1997, Ge *et al* (1997) reported a summary OR for anti-HCV positivity in HCC in China of 6.7 and other workers reported a summary OR worldwide of 11.5 (Donato *et al*, 1998), while the summary OR in the present analysis of 4.6 was significantly lower than these. This is mainly due to the fact that the ORs for anti-HCV/HCV RNA positivity in HCC of most studies after 1997 were not high, especially in the larger of the studies (Yu *et al*, 1997; Gao *et al*, 1998; Li *et al*, 1999; Ding *et al*, 2004). The studies performed before 1997 mainly used the first or second generation anti-HCV test with low sensitivity and specificity, while since then third generation anti-HCV test and RT–PCR have been widely used with improved sensitivity and specificity. The OR for HBsAg positivity and for anti-HCV/HCV RNA positivity using hospital controls in this analysis were 10.5 and 4.4, both lower than the corresponding estimates using community controls (18.0 and 4.7), probably because chronic infections by HBV and HCV were more prevalent in hospital patients than those in the general population (Donato *et al*, 1998).

The above studies did not evaluate the dual infection by HBV and HCV as in our analysis. The OR for HBsAg positivity anti-HCV/HCV RNA negativity and for HBsAg negativity anti-HCV/HCV RNA positivity in this analysis were 15.6 and 8.0, respectively. Although these indicate a strong association between each infection alone and HCC in China, the corresponding ORs worldwide of 22.5 and 17.3 were significantly higher, especially for HCV, showing that the risk is higher in western countries. In fact, the proportion of HCC patients for anti-HCV/HCV RNA positivity was 19.13% among HBsAg positive patients and 28.01% among HBsAg negative patients, and the corresponding proportions worldwide were 19.3 and 35.8% (Donato *et al*, 1998), which also supported the results. All the above results indicate that chronic infection by HBV and HCV alone were independent high risk factors for HCC.

The proportion showing positivity for both HBsAg and anti-HCV/HCV RNA in HCC patients was 13.78% in the studies overall, 12.13% in studies using hospital controls and 15.88% in studies using community controls, while the proportion in controls overall was only 1.37% (Donato *et al*, 1998). The proportion of positivity for both HBsAg and anti-HCV/HCV RNA has been reported as 6.29% in HCC patients and 0.20% in controls, and showed that the dual infection rate by HBV and HCV in China was higher than that in western countries both in cases and controls. As China is a hyperepidemic area for HBV, HCV and HCC, the above results suggested a close relation between HCC and the dual infection by HBV and HCV. Our OR for positivity for both HBsAg and anti-HCV/HCV RNA was 35.7 in the studies overall, 44.9 in studies using hospital controls and 39.5 using community controls. Donato *et al* (1998) reported a corresponding overall OR of 135, 34.6 for hospital controls and 420 using community controls, which differ appreciably from our results. The low sensitivity and specificity of anti-HCV testing in early years and low dual infection rate by HBV and HCV in western countries might account for the differences. Donato *et al* (1998) reported only 14 persons positive for both HBsAg and anti-HCV/HCV RNA in 6988 controls, whereas there were 55 ones in 4005 controls in this analysis, showing that the dual infection rate by HBV and HCV in general population in China was significantly higher than that in western countries (1.37 *vs* 0.20%, *P*<0.001).

In this analysis, the OR for positivity for both HBsAg and anti-HCV/HCV RNA was higher than the sum of the OR for HBsAg positivity anti-HCV/HCV RNA negativity and for HBsAg negativity anti-HCV/HCV RNA positivity, 35.7 *vs* 23.7 in total studies, 44.9 *vs* 17.8 in studies using hospital controls and 39.5 *vs* 31.0 in studies using community controls. All the results mentioned above indicate that the concurrent infection by HBV and HCV was associated with a much higher risk of HCC than each infection alone in China, pointing to a synergism between HBV and HCV in HCC. We did not find significant differences between the results in higher and lower incidence areas, probably owing to the diversity of risk factors for HCC in China, such as aflatoxin intake, drinking pond water, eating pickle, etc.

The different mechanisms that have been hypothesised as being associated with development of HBV- or HCV-related cancer suggest that both viruses could play an active role at different steps of the carcinogenic process when they are present together in hepatocytes. Most evidence suggests that HBV is capable of initiating the neoplastic process, while HCV could act as a promoter, and that they may be synergistic in causing HCC (Donato *et al*, 1998).

As meta-analyses are based on published studies, bias and confounding factors may be present (Egger and Smith, 1998). The interaction of other risk factors with HBV and HCV infections was not studied in this analysis. Bias and potential confounding factors may not be well controlled due to the limited details in the literature, and may therefore slightly affect the results. For more accurate results, further studies are required.

## Figures and Tables

**Table 1:** Characteristics of the case–control studies in the analysis

		**Cases**		**Controls**		
**Study**	**Area**	**Number**	**Mean age (year)**	**Number**	**Mean age (year)**	**Type of controls^a^**
Xu (1990)	Jiangsu	50	NR	50	NR	com
Gong (1993)	Guangxi	87	40	80	38	com
Zhang (1993)	Guangxi	78	NR^b^	262	NR	com
Ye (1994)	Jiangsu	110	45	220	45	com
Zhou (1994)	Guangdong, Guangxi, Hainan	104	48	208	48	com
Fan (1995)	Guangdong	64	52	128	52	com
Fan (1995)	Guangdong	72	48	128	49	com
Pan (1995)	Beijing	109	NR	109	NR	hosp
Wang (1996)	Henan	70	NR	140	NR	com
Wang (1996)	Guangdong	96	NR	144	NR	com
Guo (1997)	Neimenggu	38	49	45	48	hosp
Lu (1997)	Anhui	35	55	50	52	com
Lu (1997)	Guangxi	200	NR	60	NR	hosp
Ma (1997)	Zhejiang	31	55	17	54	hosp
Shen (1997)	Jiangsu	140	47	247	45	com
Yu (1997)	Jiangsu, Fujian, Guangxi, Hebei	340	49	350	49	hosp
Zuo (1997)	Hebei	100	49	100	47	hosp
Dai (1998)	Henan	96	NR	96	NR	hosp
Gao (1998)	Liaoning	52	54	54	55	hosp
Zhang (1998)	Henan	152	52	115	53	hosp
Cui (1999)	Jilin	112	NR	112	NR	com
Li (1999)	Jilin	100	NR	80	NR	hosp
Wang (1999)	Guangdong	100	NR	100	NR	hosp
Yang (1999)	Shanxi	98	NR	196	NR	hosp
Zhao (2000)	Anhui	87	44	100	48	com
Shi (2001)	Jiangsu	45	53	30	58	hosp
Li (2002)	Fujian	157	53	30	54	hosp
Su (2002)	Guangxi	69	41	120	43	com
Wang (2003)	Henan	53	NR	68	NR	com
Wu (2003)	Guangxi	52	43	52	45	com
Zeng (2003)	Jiangsu	100	NR	100	NR	hosp
Ding (2004)	Jiangsu	204	NR	414	NR	com

In total		3201		4005		

a Com=community-based controls; hosp=subjects hospitalised for any disease except liver disease.

b NR=not reported.

**Table 2:** Summary estimates for HBsAg and anti-HCV in total studies and according to type of controls and geographical area

	**Number of studies**	**HBsAg-positive OR (95% CI)**	**anti-HCV-positive OR (95% CI)**
Total studies	32	14.1 (10.6–18.8)	4.6 (3.6–5.9)
*Type of controls*			
Hospital	15	10.5 (6.6–16.6)	4.4 (2.9–6.6)
Community	17	18.0 (12.8–25.3)	4.7 (3.6–6.1)

*Geographical area*			
Higher incidence area	19	14.4 (10.4–19.9)	5.3 (3.8–7.4)
Lower incidence area	13	13.6 (7.7–24.0)	3.8 (2.8–5.2)

**Table 3:** Risk estimates for each HBsAg and anti-HCV combination studies using community as controls

	**HBsAg-negative anti-HCV-negative**	**HBsAg-positive anti-HCV-negative**		**HBsAg-negative anti-HCV-positive**		**HBsAg-positive anti-HCV-positive**	
**Study**	**Ca/Co^a^**	**Ca/Co**	**OR (95% CI)**	**Ca/Co**	**OR (95% CI)**	**Ca/Co**	**OR (95% CI)**
Xu (1990)	11/46	35/4	36.6 (12.9–104)	1/0	—	3/0	—
Gong (1993)	15/68	62/11	25.6 (11.9–54.9)	0/1	—	10/0	—
Zhang (1993)	12/170	40/50	11.5 (6.1–21.7)	5/31	2.3 (0.8–6.9)	21/9	33.4 (15.5–72.4)
Zhou (1994)	11/179	80/26	50.1 (27.0–92.8)	3/1	48.8 (11.5–207)	10/2	81.4 (29.6–224)
Ye (1994)	13/105	79/105	6.1 (3.3–11.1)	3/4	6.1 (1.4–25.6)	15/6	20.2 (8.0–51.2)
Fan (1995)	7/109	45/16	43.8 (19.9–96.3)	3/1	46.7 (10.1–217)	9/2	70.1 (22.2–221)
Fan (1995)	8/109	51/16	43.4 (20.2–93.3)	3/1	40.9 (5.5–306)	10/2	60.6 (20.0–184)
Wang (1996)	9/123	51/14	49.8 (23.6–105)	4/2	27.3 (5.9–127)	6/1	82.0 (22.0–306)
Wang (1996)	35/122	53/20	9.2 (5.1–16.8)	3/1	10.5 (1.6–68.7)	5/1	17.4 (3.4–90.6)
Lu (1997)	7/33	25/16	7.4 (2.8–19.7)	0/0	—	3/1	14.1 (1.9–105)
Shen (1997)	42/198	70/18	14.5 (9.2–23.0)	6/8	3.5 (1.9–6.7)	15/1	70.7 (37.8–132)
Cui (1999)	15/92	65/14	28.5 (14.0–57.8)	18/5	22.1 (8.6–57.0)	14/1	85.9 (23.5–314)
Zhao (2000)	4/86	53/11	103.6 (30.4–353)	11/3	78.8 (24.4–254)	19/0	—
Su (2002)	16/98	47/15	19.2 (9.4–39.1)	2/6	2.0 (0.4–10.7)	4/1	24.5 (4.8–125)
Wang (2003)	7/58	41/7	48.5 (18.5–127)	2/2	8.3 (1.3–52.6)	3/1	24.9 (4.2–149)
Wu (2003)	9/41	25/7	16.3 (5.7–46.5)	2/3	3.0 (0.5–19.8)	16/1	72.9 (16.1–330)
Ding (2004)	28/162	157/240	3.8 (2.5–5.8)	3/0	—	16/12	7.7 (3.6–16.6)

a Ca/Co, cases/controls.

**Table 4:** Risk estimates for each HBsAg and anti-HCV combination studies using hospital as controls

	**HBsAg-negative anti-HCV-negative**	**HBsAg-positive anti-HCV-negative**		**HBsAg-negative anti-HCV-positive**		**HBsAg-positive anti-HCV-positive**	
**Study**	**Ca/Co**	**Ca/Co**	**OR (95% CI)**	**Ca/Co**	**OR (95% CI)**	**Ca/Co**	**OR (95% CI)**
Pan (1995)	5/57	87/45	22.0 (9.8–49.4)	6/3	22.8 (5.9–88.8)	11/4	31.4 (9.3–106)
Guo (1997)	8/29	27/16	6.1 (2.3–16.1)	0/0	—	3/0	—
Lu (1997)	44/56	71//4	22.6 (9.4–54.3)	47/0	—	38/0	—
Ma (1997)	2/13	13/3	28.2 (4.9–164)	2/1	13.0 (1.0–167)	14/0	—
Yu (1997)	95/249	184/79	6.1 (4.3–8.6)	19/21	2.4 ((1.2–4.5)	42/1	110 (38.0–305)
Zuo (1997)	26/74	59/20	8.4 (4.4–16.0)	5/5	2.9 (0.8–10.3)	10/1	28.5 (6.3–129)
Dai (1998)	17/79	60/8	34.9 (15.9–76.3)	1/8	0.6 (0.2–1.7)	18/1	83.7 (22.6–309)
Gao (1998)	8/21	24/21	3.0 (1.1–8.1)	5/9	1.5 (0.4–5.8)	15/3	13.1 (3.3–51.8)
Zhang (1998)	33/101	102/10	31.2 (16.3–59.7)	3/3	3.1 (0.6–14.9)	14/1	42.9 (1.0–167)
Li (1999)	12/41	42/31	4.6 (2.1–10.1)	14/6	8.0 (3.1–20.6)	14/2	23.9 (6.3–91.0)
Wang (1999)	9/75	50/21	19.8 (9.2–42.9)	11/3	30.6 (9.6–97.0)	30/1	250 (71.2–878)
Yang (1999)	35/179	55/16	17.6 (9. 8–31.6)	2/1	10.2 (1.4–74.5)	6/0	—
Shi (2001)	13/27	27/3	18.7 (5.6–62.2)	1/0	—	4/0	—
Li (2002)	13/16	14/5	3.5 (1.0–11.9)	44/9	6.0 (2.3–16.1)	36/0	—
Zeng (2003)	22/82	70/18	14.5 (9.2–23.0)	1/1	3.7 (03–52.5)	7/0	—

**Table 5:** OR and 95% CI for each HBsAg and anti-HCV/HCV RNA combination in total studies and according to type of controls and geographical area

		**HBsAg-positive anti-HCV-negative**	**HBsAg-negative anti-HCV-positive**	**HBsAg-positive anti-HCV-positive**
	**Number of studies**	**OR (95% CI)**	**OR (95% CI)**	**OR (95% CI)**
Total studies	32	15.6 (11.5–21.3)	8.1 (5.0–13.0)	35.7 (26.2–48.5)
*Type of controls*				
Hospital	15	11.9 (7.9–17.8)	5.9 (3.1–11.4)	44.9 (27.2–74.1)
Community	17	19.9 (12.4–32.0)	11.1 (5.7–21.7)	39.5 (22.9–68.1)

*Geographical area*				
Higher incidence area	19	14.6 (9.9–21.4)	8.1 (4.5–14.7)	34.0 (23.4–49.5)
Lower incidence area	13	17.3 (10.2–29.4)	7.8 (3.4–17.8)	39.3 (22.9–67.6)

## References

[bib1] Benvegnu L, Fattovich G, Noventa F, Tremolada F, Chemello L, Cecchetto A, Alberti A (1994) Concurrent hepatitis B and C virus infection and risk of hepatocellular carcinoma in cirrhosis. A prospective study. Cancer 74: 2442–2448 7922998 10.1002/1097-0142(19941101)74:9<2442::aid-cncr2820740909>3.0.co;2-#

[bib2] Cui LH, Fang JN, Jin HZ, Quan ZY, Jin CJ (1999) An analysis of the interaction of Hepatitis virus in primary hepatocellular carcinoma in Korean of Yanbian. Chin J Prev Control Chron Non-commun Dis 7: 28–30

[bib3] Dai M, Shi Y, Zhang JY, Zhou YF, Zhang MX, He Z (1998) A study of the interaction of HBV and HCV in PHC. Henan J Oncol 11: 81–83

[bib4] DerSimonian R, Laird N (1986) Meta-analysis in clinical trials. Control Clin Trials 7: 177–188 3802833 10.1016/0197-2456(86)90046-2

[bib5] Ding BG, Fan DM, Mu LN, Yu SZ (2004) A case–control study of hepatitis B virus, hepatitis C virus and liver cancer. Chin J Epidemiol 25: 22

[bib6] Donato F, Boffetta P, Puoti M (1998) A meta-analysis of epidemiological studies on the combined effect of hepatitis B and C virus infections in causing hepatocellular carcinoma. Int J Cancer 75: 347–354 9455792 10.1002/(sici)1097-0215(19980130)75:3<347::aid-ijc4>3.0.co;2-2

[bib7] Egger MJ, Smith GD (1998) Bias in location and selection of studies. BMJ 316: 61–67 9451274 10.1136/bmj.316.7124.61 PMC2665334

[bib8] Fan XL, Peng WW, Yao JL, Zhou YP, Lu L, Zhen XC, Chen Q (1995a) Infection of HBV, HCV and HDV in hepatocellular carcinoma. Chin J Infect Dis 13: 129–132

[bib9] Fan XL, Zhou YP, Peng WW, Yao JL, Li GH, Guo RP (1995b) A case control study on relationship between hepatocellular carcinoma and infection of HCV and HBV in Guangzhou. Chin J Cancer 14: 328–330

[bib10] Gao XH, Zhang CL, Li XL, Mei H, Qi F, Zhu XY (1998) Analysis of the infection hepatitis B and C from sera of the patients suffered from primary hepatoma. J Dalian Med Univ 20: 19–21

[bib11] Ge ZX, Huang JF, Lu SC (1997) Meta-analysis of the relation between HCV infection and HCC in China: combined analysis of 19 case–control studies. Chin J Health Stat 14: 10–12

[bib12] Gong J, Yang JY, Li RC, Wang SS, Li YP, Chen KL (1993) Hepatitis C virus and primary liver cancer in a hyperendemic area of liver cancer. Chin J Public Health 12: 328–330

[bib13] Guo XL, Yan MX (1997) A study of markers of HCV and HBV in hepatocellular carcinoma. Acta Acad Med Nei Mongol 19: 58

[bib14] Li D, Cui LH, Jin DZ, Piao XX (1999) Analysis of serum hepatitis B virus and hepatitis C virus infection markers in patients with primary carcinoma of liver. J Med Sci Yanbian Univ 22: 185–188

[bib15] Li XJ, Yang HX, Wang XQ, Tang NH, Xu CS (2002) Detection of HBV and HCV in patients with primary liver cancer. J Fujian Med Univ 36: 380–381

[bib16] Llovet JM, Burroughs A, Bruix J (2003) Hepatocellular carcinoma. Lancet 362: 1907–1917 14667750 10.1016/S0140-6736(03)14964-1

[bib17] Lu CH, Hou SJ (1997) The relationship in HBV, HCV infection and cirrhosis and primary liver cancer. Clin Med 17: 17–18

[bib18] Lu YP (1997) An analysis of HBV and HCV infection of patients with primary liver cancer. Guangxi Med J 19: 144–145

[bib19] Ma ZM, Feng YZ, Chen Z (1997) HBV, HCV infection and hepatocellular carcinoma. Chin J Clin Oncol 24: 108–111

[bib20] Pan WS, Tian X, Tang SZ (1995) A case–control study of HCV infection and primary liver cancer. Chin Public Health 11: 291–294

[bib21] Parkin DM, Bray F, Ferlay J, Pisani P (2001) Estimating the world cancer burden: Globocan 2000. Int J Cancer 94: 153–156 11668491 10.1002/ijc.1440

[bib22] Robbins J, Breslow N, Greenland S (1986) Estimators of the Mantel–Haenszel variance consistent in both sparse data and large strata limiting models. Biometrics 42: 311–323 3741973

[bib23] Rothman KJ (1986) Modern Epidemiology. Boston: Little, Brown, and Company

[bib24] Shen J, Xu YC, Gao Z, Niu JY, Shen HB, Ye BF (1997) Epidemiological statocellulaudy on the etiologic synergistic interaction of HCV and HBV in the development of hepatocacinoma. Chin Natl J New Gastroenterol 14: 72–74

[bib25] Shi CJ, Zhu RM, Li XH (2001) A clinical study of relationship between hepatitis C virus and hepatocellular carcinoma. Chin J Prim Med Pharm 8: 329

[bib26] Su MH, Wu JZ, Luo GH, Huang LY, Chen MW (2002) A case–control study of relationship in HBV, HCV infection and HCC in areas with low incidences in Guangxi. Clin Foc 17: 209–210

[bib27] Tang ZY (2001) Hepatocellular carcinoma-cause, treatment and metastasis. World J Gastroenterol 7: 445–454 11819809 10.3748/wjg.v7.i4.445 PMC4688653

[bib28] Tsai JF, Jeng JE, Chang WY, Lin ZY, Tsai JH (1994) Hepatitis C viras infection among patients with chronic liver disease in an area hyperendemic for hepatitis B. Scand J Gastroenterol 29: 550–552 8079115 10.3109/00365529409092471

[bib29] Wang CX, Feng XH, Yyuan RZ, Li DS, Wang HJ, Cheng SQ (1996a) A study of the relationship in HBV, HCV infection and primary hepatocellular carcinoma. Chin J Health Lab Technol 6: 184–185

[bib30] Wang SS, Jiang PL, Pang HX, Peng GF (1999) An epidemiological study on the etiology of primary hepatocellular carcinoma in Guangzhou, Guangdong Province. Chin J Epidemiol 18: 33–36 9812479

[bib31] Wang ZF (2003) A study of the relationship between the primary carcinoma of liver and hepatitis B, C and TTV. Henan Med Res 12: 43–44

[bib32] Wang ZJ, Zhou YP, Cheng B, Liang ZN, Peng WW (1996b) An epidemiology study on the aetiology factors of primary liver cancer in Shunde city of Guangdong province. Chin J Epidemiol 17: 141–144 9208509

[bib33] Wu JZ, Su MH, Chen MW, Luo GH, Liang RX, Wei ZL, Jiang JN, Huang LY (2003) A matching case–control study on the relationship between HBV, HCV infection and HCC in endemic area of HCC in Guangxi, China. J Guangxi Med Univ 20: 313–315

[bib34] Xuezhong L, Naitoh S, Xuewen D, Song W, San Q, Li L, Hong T, Liansan Z, Bingjun L, Akahane Y (1999) Prevalence of hepatitis C virus infection in the general population and patients with liver disease in China. Hepatol Res 14: 135–143

[bib35] Xu Z, Shen FM, Xu ZY, Huang QS (1990) HCV infection and primary liver cell cancer. Tumor 10: 115

[bib36] Yang JT, Zhao HG, Zhao SF, Li PZ (1999) Prevalence of HCV and HBV infection in patients with primary hepatocellular carcinoma in Shanxi Province. Chin J Epidemiol 20: 215–217 10682498

[bib37] Yano M, Yatsuhashi H, Inoue O, Inokuchi K, Koga M (1993) Epidemiology and long term prognosis of hepatitis C virus infection in Japan. Gut 34: S13–S16 7686112 10.1136/gut.34.2_suppl.s13 PMC1373997

[bib38] Ye BF, Shen J, Xu YC, Niu JY, Chen JG, Zhang BC, Liu B, Jiang YH (1994) Etiology study on the relationship between HBV, HCV and HCC. Chin J Epidemiol 15: 131–134 7834685

[bib39] Yu SZ, Zi XL, Chen G, Li J (1997) The relationship between viral hepatitis and primary liver cancer in four areas of China. Chin J Epidemiol 18: 214–216 9812521

[bib40] Zeng JH, Dai YS, Cao MQ, Hu XK (2003) Serological markers of HCV and HBV and primary hepatocellular carcinoma. Mod Prev Med 30: 324–325

[bib41] Zhang JY, Dai M, Wang X, Lu WQ, Li DS, Zhang MX, Wang KJ, Dai LP, Han SG, Zhou YF, Zhuang H (1998) A case–control study of hepatitis B and C virus infection as risk factors for hepatocellular carcinoma in Henan, China. Int J Epidemiol 27: 574–578 9758109 10.1093/ije/27.4.574

[bib42] Zhang ZQ, Huang TR, He ZF, Yu JH, Xu QF, Huang ZD (1993) The relationship between HCV and HBV in the etiology of primary liver cancer. Guangxi Med J 15: 171–174

[bib43] Zhao JF, Yang P, Su W, Cui Z, Wang SM (2000) Hepatitis C virus infection and hepatocellular carcinoma. J Bengbu Med Coll 25: 169–170

[bib44] Zhao N, Yu SZ, Chen G, Li J, Xu HW, Wang XL, Zhen QQ, Jiang BF, Li L, Zhang AY, Lin J (1994) The combined analysis of ten case–control studies on risk factors primary hepatocarcinoma. Chin J Public Health 13: 65–68

[bib45] Zhou YP, Peng WW, Yao JL, Lu L, Li GH, Huang JF, Peng BG, Chen Q (1994) A case control study on relationship between hepatocellular carcinoma and infection of HCV and HCV. Acad J SUMS 15: 45–49

[bib46] Zuo Q, Liu YJ, Liang WT (1997) A case–control study of the synergistic action of HBV and HCV in the development of primary hepatocellular carcinoma. Chin J Public Health 16: 273–275

